# Ubiquitin Ligase Gene *OsPUB57* Negatively Regulates Rice Blast Resistance

**DOI:** 10.3390/plants14050758

**Published:** 2025-03-01

**Authors:** Jian Zhang, Qiang Du, Yugui Wu, Mengyu Shen, Furong Gao, Zhilong Wang, Xiuwen Xiao, Wenbang Tang, Qiuhong Chen

**Affiliations:** 1 College of Agronomy, Hunan Agricultural University, Changsha 410128, China; zj643961829@163.com (J.Z.); duqiang_ff@163.com (Q.D.); wuyugui1234@163.com (Y.W.); 18645244148@163.com (M.S.); gaogaofurong@163.com (F.G.); zhilongwang@126.com (Z.W.); xxwzznks@163.com (X.X.); 2 Yuelushan Laboratory, Changsha 410128, China; 3 Hunan Provincial Key Laboratory of Rice and Rapeseed Breeding for Disease Resistance, Changsha 410128, China; 4 State Key Laboratory of Hybrid Rice, Hunan Hybrid Rice Research Center, Changsha 410125, China

**Keywords:** rice, *OsPUB57*, ubiquitin ligase, *Magnaporthe oryzae*

## Abstract

The ubiquitination and degradation of proteins are widely involved in plant biotic and abiotic stress responses. E3 ubiquitin ligases play an important role in the ubiquitination of specific proteins. In this study, we identified the function of a U-box E3 ubiquitin ligase gene *OsPUB57* in rice. Expression analyses revealed that *OsPUB57* was mainly expressed in the aboveground part of rice. Drought, salt, cold, JA (jasmonic acid), PAMPs (pathogen-associated molecular patterns) or *Magnaporthe* *oryzae* treatment could significantly suppress the expression of *OsPUB57* in rice. Compared with wild-type plants, *OsPUB57*-overexpressing plants showed a decrease in resistance to *M. oryzae*, while the mutant plants exhibited an enhancement of *M. oryzae* resistance. The expression level detection indicated that *OsPUB57* negatively regulates rice blast resistance, probably by down-regulating the expression of the defense-related genes *OsPR1a* and *OsAOS2*. This study provides a candidate gene for the genetic improvement of rice blast resistance.

## 1. Introduction

The ubiquitin-proteasome system regulates protein degradation through protein polyubiquitination and 26S proteasome in eukaryotes [1,2]. In plants, this system mediates a series of life activities by degrading specific proteins, including DNA repair, tissue differentiation, signaling transduction, adversity adaptation and immune response. The process of protein ubiquitination mainly involves three enzymes, namely ubiquitin activating enzyme (E1), ubiquitin conjugating enzyme (E2) and ubiquitin ligase (E3), which are indispensable for the binding process of ubiquitin (UB) to the target protein [3,4]. E3 ubiquitin ligases can specifically recognize target proteins, which is crucial to the ubiquitin-proteasome system’s ability to specifically degrade target proteins. According to the structure and ubiquitin transfer mechanism, E3 ubiquitin ligases can be divided into two major classes including HECT (homologous to E6-associated protein C-Terminus) and RING (really interesting new gene) finger/U-box. HECT proteins can form a HECT-Ub-thioester intermediate before the ubiquitination of a substrate. RING finger/U-box proteins can directly transfer ubiquitin molecule from E2-UB intermediate to the substrate [5,6]. Generally, the U-box domain is composed of 70 amino acids with the lack of cysteine and histidine residues required for zinc chelation. It belongs to the modified RING finger domain [7,8]. U-box ubiquitin ligases are widely present in eukaryotes. There are two U-box E3 proteins in yeasts [9] and 21 U-box E3 proteins in humans [10]. In plants, 64 U-box E3 ubiquitin ligase-encoding genes are predicted in *Arabidopsis thaliana* [11], 77 in rice [12], and 121 in moso bamboo [13]. These E3 proteins with the U-box domain in plants are usually designated as PUBs (Plant U-box proteins).

PUBs are involved in the regulation of plant growth, development and various stress responses. In rice, OsPUB2 and OsPUB3 play important roles in responses to cold stress [14]. OsPUB4 was predicted to be involved in the regulation of the diurnal rhythm [15]. OsPUB9 negatively regulates the resistance to bacterial leaf blight [16]. OsSPL11/OsPUB13 and OsCIE1/OsPUB12 negatively regulate plant immunity through the ubiquitination of target proteins [17,18]. OsPUB15 influences cell death and cellular reactive oxygen species (ROS) levels [12,19]. The overexpression of OsPUB23 in rice resulted in higher plant yield due to an increase in grain size and weight [20]. OsPUB24 ubiquitinates the target protein OsBZR1 and thereby becomes a negative regulator in the BR signaling pathway [21]. OsPUB33 controls rice grain size and weight by regulating the OsNAC120-BG1 module [22]. OsPUB41 and OsPUB7 play a negative role in drought stress tolerance [23,24], while OsPUB67 plays a positive role [25]. OsPUB43 affects rice grain size and weight by modulating the transcription level of BR-responsive genes and MADS-box genes [26]. OsPUB44 positively regulates immune response and bacterial blight resistance [6]. OsPUB73 is involved in rice pollen development and disease resistance [27,28]. OsPUB75 (TUD1) affects rice growth and development and also negatively regulates drought tolerance [29,30].

The 77 PUB genes in rice can be classified into eight subfamilies based on their protein domains, with subfamily IV comprising 16 proteins that contain both the kinase domain and the U-box domain [12]. The functions of the proteins in this subfamily remain unexplored. *OsPUB57*(Os03g0424200) encodes a PUB of subfamily IV in rice. Previous studies have shown that the expression of OsPUB57 was markedly influenced by *M. oryzae,* the fungus responsible for rice blast [12]. In this study, the expression profiling of *OsPUB57* was examined under various biotic and abiotic stress treatments to assess its potential involvement in rice defense responses. Transgenic rice lines of *OsPUB57* were generated and subjected to phenotypic analyses of their rice blast resistance. The results indicate that *OsPUB57* negatively regulates resistance to rice blast. Gene expression detection results revealed that OsPUB57 may suppress rice blast resistance by regulating the expression of the disease resistance-related genes *OsPR1a* and *OsAOS2*. In conclusion, *OsPUB57* shows a potential as a candidate gene for future breeding and varietal improvement aimed at enhancing resistance to rice blast.

## 2. Results

### 2.1. Molecular Characteristics of OsPUB57 and Its Promoter

**Figure 1:**
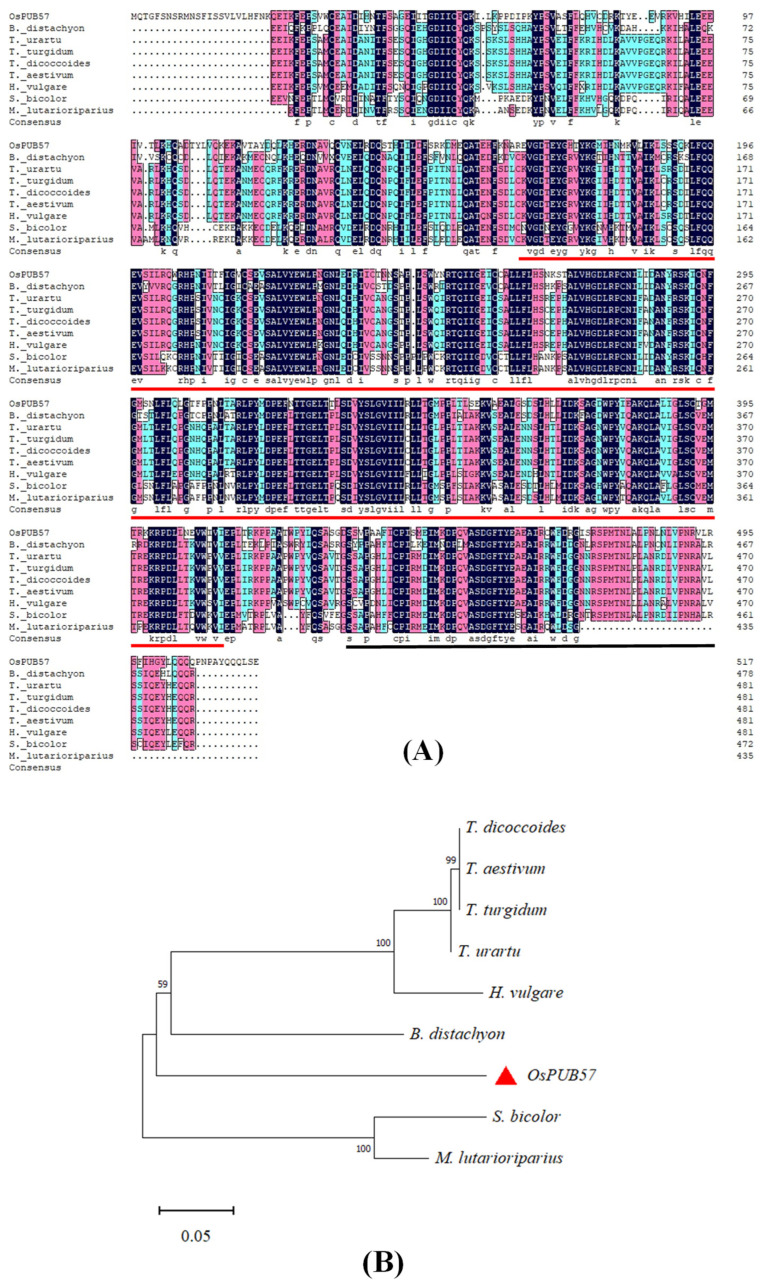
Sequence analyses of OsPUB57 and its homologous sequences. (**A**) Sequence alignment of OsPUB57 and its homologous sequences from *T. turgidum* (VAH34870.1), *T. dicoccoides* (XP_037480658.1), *T. aestivum* (XP_044458441.1), *T. urartu* (EMS46603.1), *H. vulgare* (XP_044967372.1), *B. distachyon* (XP_010230025.2), *S. bicolor* (KAG0550666.1), and *M. lutarioriparius* (CAD6211570.1). The U-box domain (black line) and kinase domain (red line) are underlined; (**B**) Phylogenetic tree of OsPUB57 and its homologs.

*OsPUB57* encodes a U-box E3 ubiquitin ligase that consists of 11 exons and 10 introns. Its full-length cDNA is 3633 bp, including 1769 bp 5′-UTR, 307 bp 3′-UTR and a 1557 bp coding region (Figure S1). OsPUB57 encodes 518 amino acids with a kinase domain and a U-box domain (Figure S1). After searching for homologous sequences, the eight most homologous sequences of OsPUB57 protein from different species (*Triticum turgidum*, *Triticum dicoccoides*, *Triticum aestivum*, *Triticum Urartu*, *Hordeum vulgare*, *Brachypodium distachyon*, *Sorghum bicolor*, and *Miscanthus lutarioriparius*) were selected for sequence alignment and phylogenetic tree analysis. The results shown in Figure 1A show that all nine sequences have the kinase domain and U-box domains, and the proportion of conserved amino acids in the nine sequences reaches 76.8%. The phylogenetic tree in Figure 1B reveals that OsPUB57 occupies a distinct branch. The homologous sequences from wheat, barley and *B*. *distachyon* were clustered into a branch. The homologous sequences from *S. bicolor* and *M. lutarioriparius* belong to a branch. In addition, when searching for the homologous sequences, no sequences from dicotyledonous plants were found in the top 100 homologous sequences.

Bioinformatics analyses revealed that *OsPUB57* has many cis-acting elements in its promoter region (Supplemental Table S2), such as a fungal elicitor response element (Box-W1), two JA signal transduction elements (CGTCA-motif and TGACG-motif), one heat stress response element (HSE), two low-temperature response elements (LTR), one drought stress response element (MES), two defense and stress response elements (TC-rich repeats), five light response elements (G-box, GT1-motif, MNF1 and Sp1(2)), three endosperm regulatory elements (GCN4-motif and Skn1-motif(2)) and so on.

### 2.2. Expression Profile of OsPUB57

**Figure 2:**
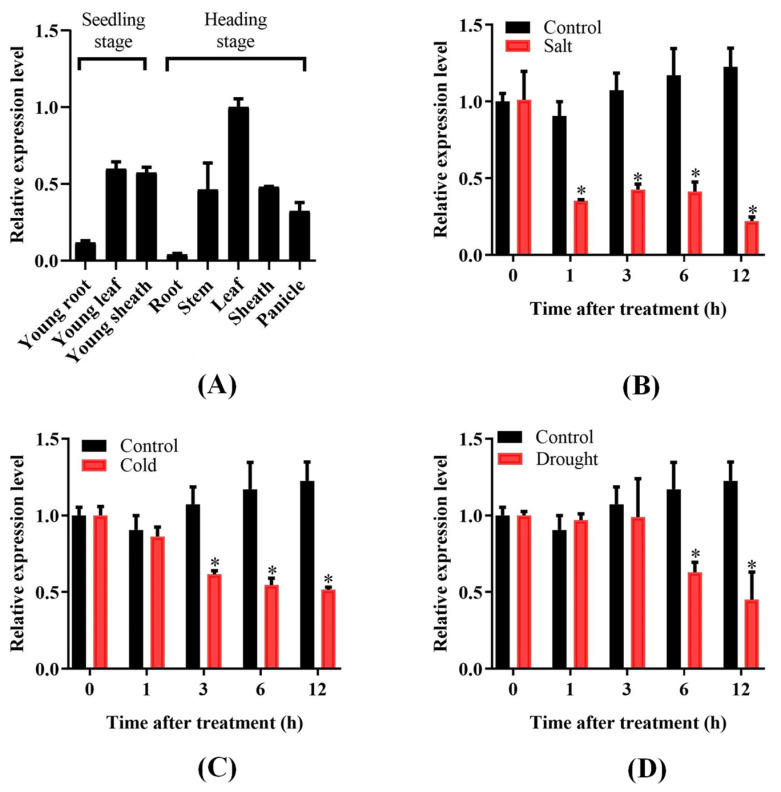
Expression profile analysis of *OsPUB57* in different rice tissues and under different stress treatments. (**A**) Expression levels of *OsPUB57* in different tissues at the seedling and heading stages; (**B**–**D**) The expression profile of *OsPUB57* under different treatments. Error bars are standard deviations based on three replicates. Analysis of significant differences in expression levels between the control and treatment samples was conducted at each time point (* represents *p* < 0.05).

The detection results of transcription levels demonstrated that OsPUB57 was more highly expressed in rice leaves or leaf sheaths than in roots at the seedling stage (Figure 2A). At the heading stage, the expression level of OsPUB57 was the highest in the leaf, followed by the stem, leaf sheath, panicle, and lowest in the root.

The expression profile of OsPUB57 in response to abiotic stresses showed that the expression level of OsPUB57 in rice seedlings decreased rapidly after salt treatment and was only about 20% after 12 h of treatment relative to that before treatment (Figure 2B). After cold treatment, the expression level of OsPUB57 gradually decreased and reached its lowest after 12 h of treatment (Figure 2C). Under drought treatment, its expression showed no significant change within the first 3 h, began to decrease significantly after 6 h of treatment and reached the lowest level at 12 h of treatment (Figure 2D).

**Figure 3:**
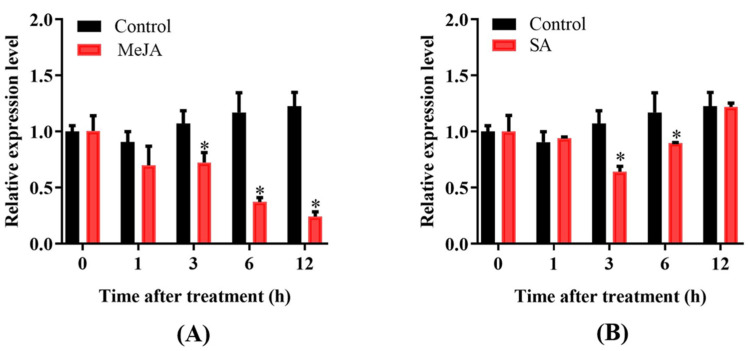
Expression profile of *OsPUB57* in rice under different phytohormone treatments. (**A**) MeJA treatments and (**B**) SA treatments. Error bars are standard deviations based on three replicates. Analyses of significant differences in expression levels between the control and treatment samples were conducted at each time point (* represents *p* < 0.05).

Under MeJA (methyl jasmonate) treatment, the expression of OsPUB57 gradually decreased and reached its lowest level at 12 h of treatment, which was only about 20% of that before treatment (Figure 3A). Under SA (salicylic acid) treatment, the expression level of OsPUB57 first decreased and then increased, and was the lowest at 3 h of treatment, which was about 65% of that before treatment (Figure 3B).

**Figure 4:**
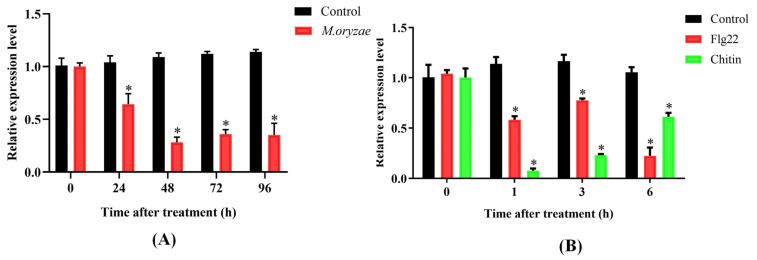
The expression level of *OsPUB57* in response to M. oryzae inoculation (**A**) and PAMP treatments (**B**). Error bars are standard deviations based on three replicates. Analyses of significant differences in expression levels between the control and treatment samples were conducted at each time point (* represents *p* < 0.05).

The expression profile of OsPUB57 in rice seedlings in response to *M. oryzae* inoculation revealed that its expression level decreased rapidly in the first 24 h after inoculation, followed by a further decrease at 48 h, and remained at a low level until 96 h (Figure 4A), indicating a significant response of *OsPUB57* to *M. oryzae* infection. When pathogens invade plants, the PTI (PAMP-triggered immunity) pathway in plants will be triggered by recognition of the PAMPs (pathogen-associated molecular patterns) of the pathogen, which in turn induces a series of signal transduction processes and changes in the expression of immunity-related genes. The expression level of OsPUB57 decreased by more than 40% at 1 h, and by about 80% at 6 h after flg22 (PAMP) treatment of the rice seedlings. Under chitin (PAMP) treatment, the expression level of OsPUB57 in rice seedlings rapidly decreased to 20% of that before treatment at 1 h of treatment, followed by slow increases, and was about 60% of that before treatment at 6 h (Figure 4B). These results indicated that OsPUB57 is very likely involved in rice’s innate immune response.

### 2.3. OsPUB57-Overexpressing Plants Showed Decreased Rice Blast Resistance

**Figure 5:**
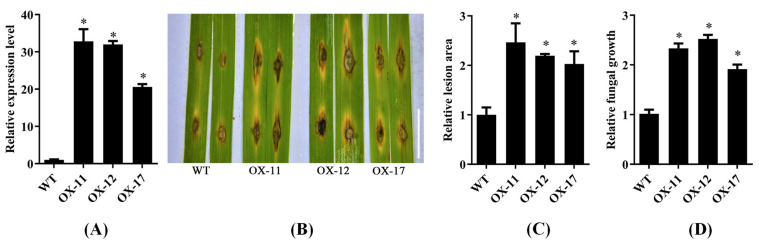
Rice blast resistance identification of *OsPUB57*-overexpressing plants (OX-11, OX-12 and OX-17) and the WT plants (Nipponbare). (**A**) The expression level of *OsPUB57*; (**B**) Rice blast resistance phenotypes. Bar = 1 cm; (**C**) Relative lesion area; (**D**) Relative fungal growth. Error bars indicate standard deviations from three replicates. Asterisks represent significant differences compared with the WT (* represents *p* < 0.05).

OsPUB57-overexpressing rice plants were created by *Agrobacterium*-mediated transgenic technology, and three overexpression lines (OX-11, OX-12 and OX-17) derived from different individual plants of T_0_ generation were obtained. Expression level detection procedures revealed that the OX-11, OX-12 and OX-17 lines had significantly higher expression levels of OsPUB57 than the wild-type plants (WT, Nipponbare) (Figure 5A). These three overexpression lines were further inoculated with *M. oryzae* for blast resistance identification. As a result, the plants of the OX-11, OX-12 and OX-17 lines showed a more susceptible phenotype than the WT plants (Figure 5B). The overexpression lines had about twofold leaf lesion areas relative to the WT (Figure 5C), as well as significantly higher relative fungal growth (Figure 5D). These results indicated that OsPUB57-overexpressing plants had significantly less rice blast resistance than the WT plants.

### 2.4. The Mutant Plants of OsPUB57 Displayed Improved Rice Blast Resistance

**Figure 6:**
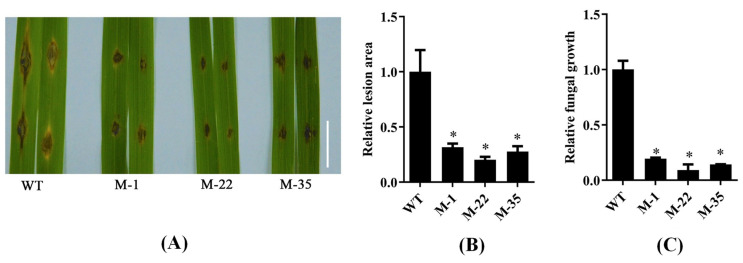
Rice blast resistance evaluation of the mutant plants (M-1, M-22 and M-35) of *OsPUB57* and the WT plants (Nipponbare). (**A**) Rice blast resistance phenotypes. Bar  = 1 cm; (**B**) Relative lesion area; (**C**) Relative fungal growth. Error bars indicate standard deviations from three replicates. Asterisks represent significant differences compared with the WT (* represents *p* < 0.05).

A target sequence (Supplemental Table S3) was selected from the DNA sequence of *OsPUB57* for gene-editing, and the corresponding transgenic rice plants were created. After PCR, sequencing and seed reproduction, three homozygous frameshift mutants, including M-1, M-22 and M-35, were selected as the mutation materials for *OsPUB57* (Supplemental Table S3). The frameshift mutations in the *OsPUB57* gene of the three mutants all led to premature termination of its translation, resulting in the loss of function of the OsPUB57 protein (Figure S2). The evaluation results of rice blast resistance (Figure 6A) showed that the mutant plants had a significantly lower disease degree than the wild-type plants (WT, Nipponbare). Figure 6B shows that the leaf lesion area of the mutants was about 20–30% that of the WT. Figure 6C reveals that the relative growth of *M. oryzae* in the leaves of mutant plants was only about 10–20% that of the WT leaves.

### 2.5. OsPUB57 May Influence the Expression of Defense-Related Genes to Regulate Rice Blast Resistance

**Figure 7:**
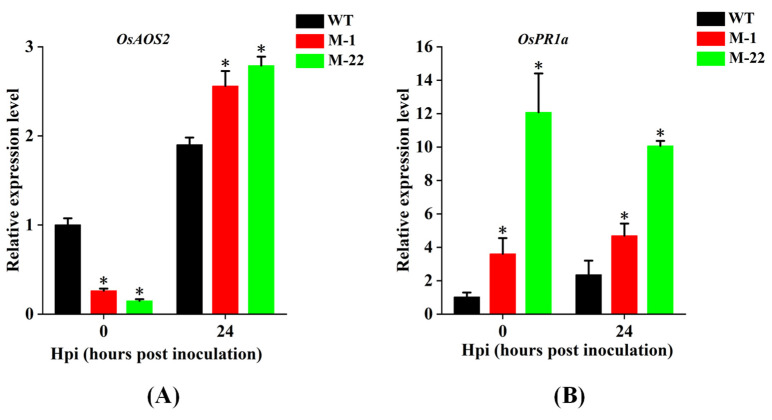
The expression levels of two defense-related genes (*OsAOS2* and *OsPR1a*) in the mutant plants of *OsPUB57* before and after inoculation with *M. oryzae*. (**A**) The expression level of *OsAOS2*. (**B**) The expression level of *OsPR1a*. Error bars indicate standard deviations from three replicates. Asterisks represent significant differences compared with the WT at each time point (* represent *p* < 0.05).

To further investigate the mechanism by which *OsPUB57* regulates rice blast resistance, the expression levels of two defense-related genes, *OsAOS2* (allene oxide synthase 2) and *OsPR1a* (pathogenesis-related 1a) [31,32,33], were measured in the mutant plants of *OsPUB57* before and after inoculation with *M. oryzae*. The results showed that, prior to inoculation with *M. oryzae* (0 h), the expression levels of *OsPR1a* in mutants M-1 and M-22 were significantly higher than those in the wild type (WT), while the expression level of *OsAOS2* in the mutants was significantly lower than that in the WT. However, 24 h after inoculation with *M. oryzae*, the expression level of both genes in the mutants was significantly higher than those in the WT. (Figure 7A,B).

## 3. Discussion

Temporal and spatial expression analyses revealed that the expression level of *OsPUB57* was the highest in leaves and extremely low in roots at both the seedling and heading stages, suggesting that *OsPUB57* should function in the aerial part but not in the underground part of rice in its natural environment. In addition, *OsPUB57* was responsive to some abiotic stress treatments. Its expression level changed greatly under salt treatment, indicating that it may be involved in regulating the salt tolerance of rice.

Usually, the innate immune response of rice will be activated upon inoculation with *M. oryzae*, through a series of signaling transduction processes named as PTI (PAMP-triggered immunity) or ETI (effector-triggered immunity), and the expression of defense-related genes will be induced for defense against pathogens [34,35]. The down-regulation of *OsPUB57* in response to PAMP (chitin and flg22) treatments or *M. oryzae* inoculation indicates that *OsPUB57* may be a negative regulator in the PTI pathway or in both the PTI and ETI pathways of rice. This speculation was also confirmed by the results of *M. oryzae* inoculation on *OsPUB57*-overexpressing plants and mutant plants. Disease resistance phenotype and statistics results of lesion areas or fungal growth revealed that *OsPUB57* negatively regulates rice blast resistance, and its mutation can improve rice blast resistance. Downstream of ETI or PTI, the activation of complex phytohormone signaling networks is crucial for stimulating the plant’s immune signaling network. SA and JA play key roles in plant signaling networks involved in local or systemic defense responses against multiple pathogens [36,37,38]. Either JA or SA treatment would induce the expression of the pathogenesis-related (PR) gene *OsPR1* in rice. Previous research has confirmed that the active form of the NPR1 (non-expressor of pathogenesis-related genes 1) protein (SA receptor) interacts with the TGACG-binding factor (TGA) transcription factor family protein, which could bind to the cis-elements of the *PR1* promoter to regulate the expression of *PR1* [39]. *OsPR1* serves as an important marker for systemic acquired resistance (SAR) [33]. In this study, both before and after inoculation with *M. oryzae*, the expression level of *OsPR1a* in the mutant plants of *OsPUB57* was significantly higher than that in the wild-type plants. This played a positive role in enhancing the disease resistance of the mutant plants. *OsAOS2*, a key gene in the JA synthesis pathway [31,40], showed significantly lower expression levels in the mutant plants before *M. oryzae* inoculation, while its expression level in the mutant plants was significantly higher than that in the wild-type plants after inoculation. *OsAOS2* was strongly induced in the mutant plants after *M. oryzae* inoculation. These results suggest that in the absence of *M. oryzae* infection, the mutation of *OsPUB57* in rice may lead to a decrease in JA synthesis. However, once the rice plants with mutated *OsPUB57* are infected with *M. oryzae*, they are able to induce JA synthesis more strongly through the activation of a certain pathway to resist the disease. The specific regulatory mechanism underlying this response needs further research. Previous studies have found that after being inoculated with *M. oryzae*, the expression level of *OsPUB57* only increased in the rice plants carrying the *Pi9*-resistant gene, while it decreased in rice plants without *Pi9* [12]. This is consistent with the results on Nipponbare (NPB) in this study, indicating that *OsPUB57* may play different roles in different immune pathways of PTI and ETI in rice.

*OsPUB57* was previously reported to have E3 ubiquitin ligase activity [12]. E3 ubiquitin ligase usually performs its biological function by mediating the ubiquitination of a specific substrate. Some PUBs in rice have been revealed to have biological functions, and some specific ubiquitination substrates have been identified. Among the 77 PUBs of rice, OsPUB9, OsCIE1(OsCERK1-interacting-E3 1)/OsPUB12, OsPUB73, OsPUB15, OsPUB44 and SPL11 (SQUAMOSA promoter binding protein-like 11)/PUB13 have been found to be involved in rice’s innate immune system. OsPUB73 positively regulates rice resistance against *M. oryzae* and *Xoo* by interacting with OsVQ25 (valine-glutamine (VQ) motif-containing protein 25) and promoting its degradation via the ubiquitin-26S proteasome pathway. OsVQ25 plays an important role in balancing rice immunity and growth through interaction with OsPUB73 and a transcription factor OsWRKY53 (tryptophan (W)-arginine (R)-lysine (K)-tyrosine (Y) 53) [28]. Rice U-box E3 ubiquitin ligase SPL11 ubiquitinated and degraded a Rho GTPase-activating protein SPIN6 (SPL11-interacting protein 6) to regulate the activity of small GTPase OsRac1 (Ras-related C3 botulinum toxin substrate 1) in rice cells, which could transfer the defense signals from SPL11 to OsRac1 to regulate plant cell death (PCD) and innate immunity [17]. OsPUB15 directly interacted with the kinase domain of PID2K and regulated PCD and blast disease resistance. PID2K can phosphorylate OsPUB15, and only the phosphorylated form of OsPUB15 has E3 ligase activity [2]. When pathogens infect plants, their effector proteins are delivered to host cells to suppress plant immunity. It is interesting that OsPUB44 is the target of an effector (*XopP_Xoo_*) of the rice pathogen *Xanthomonas oryzae* pv. *oryzae. XopP_Xoo_* directly interacts with the U-box domain of OsPUB44 and inhibits its ligase activity to suppress PGN- and chitin-triggered immunity (PTIs) and disease resistance [6]. OsCIE1/OsPUB12 ubiquitinates and curtails the kinase activity of OsCERK1 (chitin elicitor receptor kinase 1) at a basal level during homeostasis, serving as a molecular brake that prevents OsCERK1-mediated autoimmunity. It is unusual that OsCIE1-mediated ubiquitination of OsCERK1 does not mark OsCERK1 for proteasomal degradation but instead suppresses its kinase activity. In the presence of pathogens, this ubiquitination is blocked, which releases the brake and enables the full activation of OsCERK1-mediated immune signaling cascades that are required to protect plant cells from infection [18]. Determination of the ubiquitination target and the upstream regulator of OsPUB57 will be the key to revealing its specific regulatory mechanism in rice immunity.

Some proteins have been predicted to be potential interactors with OsPUB57 in the STRING database (https://cn.string-db.org/ (accessed on 10 November 2024)). Function annotation results indicated that these proteins included NB-ARC domain containing protein (Os01g0314700), protein kinases (Os02g0281000 and Os01g0872800), E3 ligase (Os10g0466300), 1-acyl-sn-glycerol-3-phosphate acyltransferase (Os10g0497100), phospholipid/glycerol acyltransferase (Os04g0625200) and so on. NB-ARC proteins are well-known for their involvement in disease resistance [41]. An activated form of the NB-ARC protein RLS1 functions with cysteine-rich receptor-like protein RMC to modulate the oxidative state, cell death process, and associated immunity responses in rice [42]. Protein kinases have been shown in multiple studies to play crucial roles in regulating rice disease resistance. Overexpression of the calcium-dependent protein kinase OsCPK4 in rice enhanced the resistance to *M. oryzae* [43]. The protein kinase OsCIPK31 (CBL-interacting protein kinase 31), in conjunction with OsCBL2 (calcineurin B-like 2) and OsAKT1L (AKT1-like), forms the CBL2-CIPK31-AKT1L signaling pathway, collectively regulating rice blast resistance [44]. The predicted interacting protein encoded by *Os01g0872800* is the protein kinase OsPdk2, which was previously reported to regulate basal disease resistance through the OsOxi1 (oxidative signal-inducible 1)-OsPti1a phosphorylation cascade in rice [45]. Further research is needed to determine whether OsPUB57 regulates rice blast resistance by interacting with some of these proteins.

## 4. Materials and Methods

### 4.1. Materials and Growth Conditions

The japonica rice variety Nipponbare (NPB) was used for various treatments, expression level detection and genetic transformation in this study. Dehulled rice seeds were sequentially grown on 1/2 MS medium for seven days and in Hoagland nutrient solution for three weeks under controlled photoperiodic conditions (14 h light, 28 °C/10 h dark, 25 °C), and then the obtained seedlings were used for various treatments, *M. oryzae* inoculation, and the expression level detection of *OsPUB57* in different tissues at the seedling stage. In addition, rice seedlings cultured under the same conditions were transplanted to a field to determine the expression level of *OsPUB57* in different tissues at the heading stage.

### 4.2. RNA Extraction and qRT-PCR Amplification

The total RNA of rice tissues was extracted using the TRIzol reagent (Carlsbad, CA, USA, Invitrogen, 15596026). First-strand cDNA was synthesized via a reverse transcription reaction of 2 µg of total RNA with a reverse transcription kit (Waltham, MA, USA, Thermo Scientific, K1682). The amplification reagent used in qRT-PCR was TB Green Premix Ex Taq (Dalian, China, TaKaRa, RR420L). The total volume of the reaction system was 20 µL, and the reaction apparatus was an Applied Biosystems Stepone^TM^ Real-Time PCR System (Waltham, MA, USA, Thermo Fisher, ABI Step One). The reaction program of qRT-PCR was as follows: 95 °C for 1 min, followed by 40 cycles at 95 °C for 5 s, 60 °C for 30 s. The internal reference gene used for the detection of the relative expression levels of rice genes was the *ubiquitin* (Os03g0234200) of rice [46]. The relative expression levels of rice genes were calculated by the 2 ^−∆∆Ct^ method using Excel software [47,48]. A *t*-test was employed to assess the significance of differences using SPSS 24, and Origin 2021 Prism was utilized for plotting.

All of the primers used in this study were synthesized by Sangon Biotech (Shanghai) Co., Ltd. (Shanghai, China). The sequences and functions of the primers are presented in Supplemental Table S1.

### 4.3. Vector Construction and Genetic Transformation of Rice

The full-length CDS of *OsPUB57* was amplified from rice whole cDNA samples. An overexpression vector of *OsPUB57* (pCXUN-OsPUB57) was constructed using the zero-background T-Vector system [49]. A 20-bp fragment was selected from the genome sequence of *OsPUB57* as the target sequence for gene-editing, and the corresponding adapter primers were designed to construct the CRISPR/Cas9 gene-editing vector [50]. The overexpression vector and gene-editing vector were genetically transformed into rice Nipponbare by Agrobacterium-mediated transgenic technology to obtain the overexpression and gene-editing plants of *OsPUB57*. The genetic transformation process was completed at Wuhan Towin Biotechnology Company Limited (Wuhan, Hubei, China).

### 4.4. Cultivation of Rice Blast Fungus

*M. oryzae* race RO1-1 was grown on oatmeal medium and cultivated in the dark at 25 °C for 1 to 2 days. When the fungi were germinated, the medium plates were moved to 25 °C for continuous light cultivation until the plates were covered with mycelia, then irradiated with blue-black light for 1 to 2 days to induce conidia. Finally, the conidia were collected.

### 4.5. Inoculation of Rice Blast Fungus

The conidial suspension (0.05% tween-20) of *M. oryzae* was prepared at a concentration of 5 × 10^5^ conidia ml-1. Freshly isolated leaves from individual rice lines were randomly selected, punched with two holes per leaf, and then placed in a petri dish containing moist filter paper wetted with 0.1% 6-benzylaminopurine (6-BA). About 5 µL of conidial suspension was added to each hole in the leaves, and then the petri dish was sealed and moved into an incubator with high humidity at 25 °C, followed by cultivation for one day in the dark. After that, the photoperiod was changed to 14 h light/10 h dark, which continued for 5–7 days. Finally, the blast resistance of the rice materials was evaluated. To determine the relative lesion area, the lesion area of each sample was first measured using ImageJ (v1.8.0.345). The lesion area of the wild-type material (WT), denoted as M, was set to 1. The relative lesion area of each sample was then calculated using the formula M0/M, where M0 represents the lesion area of each individual sample. Based on previous literature [51], to detect the relative fungal growth, DNA was extracted from the infected tissues of each sample, treated with RNase A to remove RNA, and then subjected to a DNA-based qPCR assay. The threshold cycle value (C_T_) of *M. oryzae Pot2* DNA and the C_T_ of rice genomic *ubiquitin* DNA was measured, and the C_T_ of *Ubq* was subtracted from the C_T_ of *Pot2*. Relative fungal growth was then calculated as a ratio (Mo-*Pot2*/Os-*Ubq*) represented by the equation E^CT(Os-*UBQ*) − CT(Mo-*Pot2*)^, in which the amplification efficiency, E, is 2 for the primer pairs designed for the respective genes. All inoculation experiments were repeated more than three times.

### 4.6. Various Treatments and Samplings

Rice seedlings were planted with a Hoagland nutrient solution containing 100 mM NaCl for salt stress treatment at 28 °C under light conditions [52]. Cold treatment was performed with seedlings grown in a Hoagland nutrient solution at 4 °C under light conditions [53]. Drought stress treatment was performed on rice seedlings according to the previously reported method [54]. Seedlings grown in a Hoagland nutrient solution under normal conditions were used as the control. Rice seedlings were transferred to Hoagland nutrient solutions containing 100 µM MeJA or 100 µM SA, respectively, for hormone treatments at 28 °C under light conditions [55,56]. Rice seedlings grown in a Hoagland nutrient solution without hormone were used as the control. For all of these treatments, rice samples with stems and leaves were collected at 0, 1, 3, 6 and 12 h of treatment, respectively.

For the *M. oryzae* treatment, the conidial suspension (0.05% tween-20) of *M. oryzae* was prepared at a concentration of 5 × 10^5^ conidia mL^−1^ and sprayed on rice seedlings. Then, the rice seedlings were treated in the dark for 24 h at 25 °C under high humidity. After that, the rice seedlings were put into an incubator at high humidity under controlled photoperiodic conditions (14 h light, 28 °C/10 h dark, 25 °C). Rice samples with stems and leaves were collected at 0, 24, 48, 72 and 96 h after the conidia spray.

The rice seedlings were planted with a Hoagland nutrient solution containing 100 ng/L flg22 or 8 ng/L chitin for PAMP treatment. Rice seedlings grown in a Hoagland nutrient solution without flg22 or chitin were used as the control. Rice samples with stems and leaves were collected at 0, 1, 3 and 6 h of treatment.

RNA was extracted from every sample above, and the relative expression level of *OsPUB57* was detected after reverse transcription. Significant differences were analyzed by comparing the control and treatment samples at each time point. The effectiveness of various treatments was verified by detecting the expression levels of the reported responsive marker genes through qRT-PCR. These genes included *OsTSD2* (tumorous shoot development2) for drought and salt treatments [57], *OsDREB1A* (dehydration-responsive element binding factor 1A) for cold treatment [58], *OsWrky45* (tryptophan (W)-arginine (R)-lysine (K)-tyrosine (Y) 45) for SA treatment [59], *OsJAmyb* (a JA-inducible MYB transcription factor) for JA treatment [59], *OsPAL1* (phenylalanine ammonia lyase 1) for PAMP treatments [60] and *OsHLP1* (HVA22-like protein 1) for rice blast fungus treatment [61]. The detection results of the expression levels of these marker genes indicated that the treatments successfully induced the relevant stress responses in this study (Figures S3–S8).

### 4.7. Bioinformatics Analysis

The homologous sequences of OsPUB57 were searched using blastP in the NCBI database (https://blast.ncbi.nlm.nih.gov/Blast.cgi (accessed on 28 November 2024)), with the search parameters set to the defaults. The sequences with the highest homology in eight different species were selected from the top 50 homologous sequences for sequence alignment and phylogenetic tree analysis. Phylogenetic tree analysis was conducted by the neighbor-joining method of the MEGA (v11) software (bootstrap replicates were 1000). Multiple alignment of amino acid sequences was performed using the DNAman (v8) software. The 2000 bp genome sequence upstream of the start codon (ATG) of *OsPUB57* was regarded as its promoter sequence, and the cis-acting element analysis of the promoter was performed in the PlantCARE database.

## 5. Conclusions

*OsPUB57* is a U-Box E3 ubiquitin ligase gene. It is mainly expressed in the aboveground part of rice. Drought, salt, cold, MeJA, PAMPs (pathogen-associated molecular patterns) or *M. oryzae* treatment on significantly suppressed the expression of *OsPUB57* in rice. The mutation of *OsPUB57* notably enhanced rice blast resistance by upregulating the disease resistance-related genes *OsPR1a* and *OsAOS2*, while the overexpression of *OsPUB57* in rice significantly reduced rice blast resistance. This indicates that OsPUB57 negatively regulates rice blast resistance. The predicted interacting proteins of OsPUB57 include some proteins that may be involved in the plant immune system. Future work should focus on verifying whether these proteins are real interacting proteins and ubiquitination substrates of OsPUB57 and whether these proteins are involved in the regulation of rice disease resistance.

## Data Availability

The datasets generated during the current study are available from the corresponding authors on reasonable request.

## Supplementary Materials

The following supporting information can be downloaded at: https://www.mdpi.com/article/10.3390/plants14050758/s1, Table S1. Primer sequences used in this study. Table S2. The *cis*-acting elements on the promoter region of *OsPUB57*. Table S3. The genotypes of target sites in three mutant lines of *OsPUB57*. The blue colored nucleotides indicate the target sequence of wild type. The red colored nucleotides indicate the protospacer adjacent motif (PAM). The dotted lines are the base deletions. A bold base represents the inserted base. Figure S1. Schematic structure of *OsPUB57*. Figure S2. The amino acid sequence comparison of OsPUB57 in Nipponbare (NPB) and three mutants (M-1, M-22 and M-35). Figure S3. The transcriptional expression level of *OsTSD2* in rice under salt and drought treatments. Note: Asterisks represent significant differences compared with the control. * represents *p* < 0.05, ** represents *p* < 0.01. The same below. Figure S4. The transcriptional expression level of *OsDREB1A* in rice under cold treatment. Figure S5. The transcriptional expression level of *OsWrky45* in rice under SA treatment. Figure S6. The transcriptional expression level of *OsJAmyb* in rice under JA treatment. Figure S7. The transcriptional expression level of *OsPAL1* in rice under PAMP (flg22 or chitin) treatments. Figure S8. The transcriptional expression level of *OsHLP1* in rice after *M. oryzae* inoculation.

## References

[1] Mukhopadhyay D., Riezman H. (2007). Proteasome-independent functions of ubiquitin in endocytosis and signaling. Science.

[2] Wang J., Qu B., Dou S., Li L., Yin D., Pang Z., Zhou Z., Tian M., Liu G., Xie Q. (2015). The E3 ligase *OsPUB15* interacts with the receptor-like kinase *PID2* and regulates plant cell death and innate immunity. BMC Plant Biol..

[3] Callis J. (2014). The ubiquitination machinery of the ubiquitin system. Arab. Book.

[4] Cho S.K., Ryu M.Y., Kim J.H., Hong J.S., Oh T.R., Kim W.T., Yang S.W. (2017). RING E3 ligases: Key regulatory elements are involved in abiotic stress responses in plants. BMB Rep..

[5] Berndsen C.E., Wolberger C. (2014). New insights into ubiquitin E3 ligase mechanism. Nat. Struct. Mol. Biol..

[6] Ishikawa K., Yamaguchi K., Sakamoto K., Yoshimura S., Inoue K., Tsuge S., Kojima C., Kawasaki T. (2014). Bacterial effector modulation of host E3 ligase activity suppresses PAMP-triggered immunity in rice. Nat. Commun..

[7] Aravind L., Koonin E.V. (2000). The U-box is a modified RING finger–A common domain in ubiquitination. Curr. Biol..

[8] Borden K.L., Freemont P.S. (1996). The RING finger domain: A recent example of a sequence-structure family. Curr. Opin. Struct. Biol..

[9] Koegl M., Hoppe T., Schlenker S., Ulrich H.D., Mayer T.U., Jentsch S. (1999). A novel ubiquitination factor, E4, is involved in multiubiquitin chain assembly. Cell.

[10] Ohi M.D., Vander Kooi C.W., Rosenberg J.A., Chazin W.J., Gould K.L. (2003). Structural insights into the U-box, a domain associated with multi-ubiquitination. Nat. Struct. Biol..

[11] Mudgil Y., Shiu S.H., Stone S.L., Salt J.N., Goring D.R. (2004). A large complement of the predicted arabidopsis ARM repeat proteins are members of the U-box E3 ubiquitin ligase family. Plant Physiol..

[12] Zeng L.R., Park C.H., Venu R.C., Gough J., Wang G.L. (2008). Classification, expression pattern, and E3 ligase activity assay of rice U-box containing proteins. Mol. Plant.

[13] Zhou J., Hu Y.P., Li J., Yu Z.Y., Guo Q.R. (2021). Genome-wide identification and expression analysis of the plant U-box protein gene family in Phyllostachys edulis. Front. Genet..

[14] Byun M.Y., Cui L.H., Oh T.K., Jung Y.J., Lee A., Park K.Y., Kang B.G., Kim W.T. (2017). Homologous U-box E3 Ubiquitin Ligases *OsPUB2* and *OsPUB3* Are Involved in the Positive Regulation of Low Temperature Stress Response in Rice (*Oryza sativa* L.). Front. Plant Sci..

[15] Yoo Y.H., Jiang X., Jung K.H. (2020). An abiotic stress responsive U-box E3 ubiquitin ligase is involved in OsGI-mediating diurnal rhythm regulating Mechanism. Plants.

[16] Kim M.S., Le V.T., Jung Y.J., Kang K.K., Cho Y.G. (2024). *OsPUB9* Gene Edited by CRISPR/Cas9 Enhanced Resistance to Bacterial Leaf Blight in Rice (*Oryza sativa* L.). Int. J. Mol. Sci..

[17] Liu J., Park C.H., He F., Nagano M., Wang M., Bellizzi M., Zhang K., Zeng X., Liu W., Ning Y. (2015). The RhoGAP SPIN6 associates with SPL11 and OsRac1 and negatively regulates programmed cell death and innate immunity in rice. PLoS Pathog..

[18] Wang G., Chen X., Yu C., Shi X., Lan W., Gao C., Yang J., Dai H., Zhang X., Zhang H. (2024). Release of a ubiquitin brake activates OsCERK1-triggered immunity in rice. Nature.

[19] Park J.J., Yi J., Yoon J., Cho L.H., Ping J., Jeong H.J., Cho S.K., Kim W.T., An G. (2011). *OsPUB15*, an E3 ubiquitin ligase, functions to reduce cellular oxidative stress during seedling establishment. Plant J..

[20] Li H., Wang Y., Qiao W., Zhu Z., Wang Z., Tian Y., Liu S., Wan J., Liu L. (2024). Identification of a novel locus *qGW12/OsPUB23* regulating grain shape and weight in rice (*Oryza sativa* L.). Theor. Appl. Genet..

[21] Min H.J., Cui L.H., Oh T.R., Kim J.H., Kim T.W., Kim W.T. (2019). OsBZR1 turnover mediated by OsSK22-regulated U-box E3 ligase *OsPUB24* in rice BR response. Plant J..

[22] Xie Z., Sun Y., Zhan C., Qu C., Jin N., Gu X., Huang J. (2024). The E3 ligase *OsPUB33* controls rice grain size and weight by regulating the OsNAC120-BG1 module. Plant Cell.

[23] Seo D.H., Lee A., Yu S.G., Cui L.H., Min H.J., Lee S.E., Cho N.H., Kim S., Bae H., Kim W.T. (2021). *OsPUB41*, a U-box E3 ubiquitin ligase, acts as a negative regulator of drought stress response in rice (*Oryza sativa* L.). Plant Mol. Biol..

[24] Kim M.S., Ko S.R., Jung Y.J., Kang K.K., Lee Y.J., Cho Y.G. (2023). Knockout Mutants of *OsPUB7* Generated Using CRISPR/Cas9 Revealed Abiotic Stress Tolerance in Rice. Int. J. Mol. Sci..

[25] Qin Q., Wang Y., Huang L., Du F., Zhao X., Li Z., Wang W., Fu B. (2020). A U-box E3 ubiquitin ligase *OsPUB67* is positively involved in drought tolerance in rice. Plant Mol. Biol..

[26] Wu Q., Liu Y., Huang J. (2022). CRISPR-Cas9 mediated mutation in *OsPUB43* improves grain length and weight in rice by promoting cell proliferation in spikelet hull. Int. J. Mol. Sci..

[27] Chen L., Deng R., Liu G., Jin J., Wu J., Liu X. (2019). Cytological and transcriptome analyses reveal *OsPUB73* defect affects the gene expression associated with tapetum or pollen exine abnormality in rice. BMC Plant Biol..

[28] Hao Z., Tian J., Fang H., Fang L., Xu X., He F., Li S., Xie W., Du Q., You X. (2022). A VQ-motif-containing protein fine-tunes rice immunity and growth by a hierarchical regulatory mechanism. Cell Rep..

[29] Hu X., Qian Q., Xu T., Zhang Y., Dong G., Gao T., Xie Q., Xue Y. (2013). The U-box E3 ubiquitin ligase *TUD1* functions with a heterotrimeric G α subunit to regulate Brassinosteroid-mediated growth in rice. PLoS Genet..

[30] Sun Y., Gu X., Qu C., Jin N., Qin T., Jin L., Huang J. (2024). OsPUB75-OsHDA716 mediates deactivation and degradation of *OsbZIP46* to negatively regulate drought tolerance in rice. Plant Physiol..

[31] Mei C., Qi M., Sheng G., Yang Y. (2006). Inducible overexpression of a rice allene oxide synthase gene increases the endogenous jasmonic acid level, *PR* gene expression, and host resistance to fungal infection. Mol. Plant Microbe Interact..

[32] Ma J., Morel J.B., Riemann M., Nick P. (2022). Jasmonic acid contributes to rice resistance against *Magnaporthe oryzae*. BMC Plant Biol..

[33] Kumari D., Prasad B.D., Dwivedi P., Sahni S., Kumar M., Alamri S., Adil M.F., Alakeel K.A. (2024). Comprehensive analysis of transcription factor binding sites and expression profiling of rice pathogenesis related genes (*OsPR1*). Front. Plant Sci..

[34] Ngou B.P.M., Ding P., Jones J.D.G. (2022). Thirty years of resistance: Zig-zag through the plant immune system. Plant Cell..

[35] Yuan M.H., Ngou B.P.M., Ding P.T., Xin X.F. (2021). PTI-ETI crosstalk: An integrative view of plant immunity. Curr. Opin. Plant Biol..

[36] Yang Y.X., Ahammed G.J., Wu C.J., Fan S.Y., Zhou Y.H. (2015). Crosstalk among jasmonate, salicylate and ethylene signaling pathways in plant disease and immune responses. Curr. Protein Pept. Sci..

[37] Zhou J.M., Zhang Y.L. (2020). Plant immunity: Danger perception and signaling. Cell.

[38] La Spada F., Stracquadanio C., Riolo M., Pane A., Cacciola S.O. (2020). Trichoderma Counteractsthe Challenge of Phytophthora nicotianae Infections on Tomato by Modulating Plant Defense Mechanisms and the Expression of Crinkler, Necrosis-Inducing Phytophthora Protein 1, and Cellulose-Binding Elicitor Lectin Pathogenic Effectors. Front. Plant Sci..

[39] Li N., Han X., Feng D., Yuan D., Huang L.J. (2019). Signaling Crosstalk between Salicylic Acid and Ethylene/Jasmonate in Plant Defense: Do We Understand What They Are Whispering?. Int. J. Mol. Sci..

[40] Ken H., Moritoshi I. (2004). Phytochrome-Mediated Transcriptional Up-regulation of ALLENE OXIDE SYNTHASE in Rice Seedlings. Plant Cell Physiol..

[41] Takken F.L., Albrecht M., Tameling W.I. (2006). Resistance proteins: Molecular switches of plant defence. Curr. Opin. Plant Biol..

[42] Wang Y., Teng Z., Li H., Wang W., Xu F., Sun K., Chu J., Qian Y., Loake G.J., Chu C. (2023). An activated form of NB-ARC protein RLS1 functions with cysteine-rich receptor-like protein RMC to trigger cell death in rice. Plant Commun..

[43] Bundó M., Coca M. (2016). Enhancing blast disease resistance by overexpression of the calcium-dependent protein kinase *OsCPK4* in rice. Plant Biotechnol. J..

[44] Lin Q.J., Kumar V., Chu J., Li Z.M., Wu X.X., Dong H., Sun Q., Xuan Y.H. (2021). CBL-interacting protein kinase 31 regulates rice resistance to blast disease by modulating cellular potassium levels. Biochem. Biophys. Res. Commun..

[45] Matsui H., Miyao A., Takahashi A., Hirochika H. (2010). Pdk1 kinase regulates basal disease resistance through the OsOxi1-OsPti1a phosphorylation cascade in rice. Plant Cell Physiol..

[46] Kong W., Zhong H., Deng X., Gautam M., Gong Z., Zhang Y., Zhao G., Liu C., Li Y. (2019). Evolutionary Analysis of *GH3* Genes in Six Oryza Species/Subspecies and Their Expression under Salinity Stress in *Oryza sativa* ssp. japonica. Plants.

[47] Wang D., Wan H., Zhang S., Yu J. (2009). γ-MYN: A new algorithm for estimating Ka and Ks with consideration of variable substitution rates. Biol. Direct..

[48] Boubakri H., Chihaoui S., Najjar E., Gargouri M., Barhoumi F., Jebara M. (2021). Genome-wide analysis and expression profiling of H-type Trx family in Phaseolus vulgaris revealed distinctive isoforms associated with symbiotic N2-fixing performance and abiotic stress response. Plant Physiol..

[49] Chen S., Songkumarn P., Liu J., Wang G.L. (2009). A versatile zero background T-vector system for gene cloning and functional genomics. Plant Physiol..

[50] Ma X., Zhang Q., Zhu Q., Liu W., Chen Y., Qiu R., Wang B., Yang Z., Li H., Lin Y. (2015). A robust CRISPR/Cas9 system for convenient high-efficiency multiplex genome editing in monocot and dicot plants. Mol. Plant.

[51] Park C.H., Chen S., Shirsekar G., Zhou B., Khang C.H., Songkumarn P., Afzal A.J., Ning Y., Wang R., Bellizzi M. (2012). The Magnaporthe oryzae effector AvrPiz-t targets the RING E3 ubiquitin ligase *APIP6* to suppress pathogen associated molecular pattern-triggered immunity in rice. Plant Cell.

[52] Chourey K., Ramani S., Apte S.K. (2003). Accumulation of LEA proteins in salt (NaCl) stressed young seedlings of rice (*Oryza sativa* L.) cultivar Bura Rata and their degradation during recovery from salinity stress. Plant Physiol..

[53] Zhai M., Chen Y., Pan X., Chen Y., Zhou J., Jiang X., Zhang Z., Xiao G., Zhang H. (2024). OsEIN2-OsEIL1/2 pathway negatively regulates chilling tolerance by attenuating *OsICE1* function in rice. Plant Cell Environ..

[54] Ning Y., Jantasuriyarat C., Zhao Q., Zhang H., Chen S., Liu J., Liu L., Tang S., Park C.H., Wang X. (2011). The SINA E3 ligase *OsDIS1* negatively regulates drought response in rice. Plant Physiol..

[55] Kapoor R., Kumar G., Arya P., Jaswal R., Jain P., Singh K., Sharma T.R. (2019). Genome-Wide Analysis and Expression Profiling of Rice Hybrid Proline-Rich Proteins in Response to Biotic and Abiotic Stresses, and Hormone Treatment. Plants.

[56] Yamada S., Kano A., Tamaoki D., Miyamoto A., Shishido H., Miyoshi S., Taniguchi S., Akimitsu K., Gomi K. (2012). Involvement of *OsJAZ8* in jasmonate-induced resistance to bacterial blight in rice. Plant Cell Physiol..

[57] Fang C., Li K., Wu Y., Wang D., Zhou J., Liu X., Li Y., Jin C., Liu X., Alejandro J.L.M. (2019). *OsTSD2*-mediated cell wall modification affects ion homeostasis and salt tolerance. Plant.

[58] Dubouzet J.G., Sakuma Y., Ito Y., Kasuga M., Dubouzet E.G., Miura S., Seki M., Shinozaki K., Yamaguchi-Shinozaki K. (2003). *OsDREB* genes in rice, *Oryza sativa* L., encode transcription activators that function in drought- high-salt- and cold-responsive gene expression. Plant J..

[59] Xie J., Yang F., Xu X., Peng Y., Ji H. (2022). Salicylic Acid, Jasmonate, and Ethylene Contribute to Rice Defense Against White Tip Nematodes *Aphelenchoides besseyi*. Front. Plant Sci..

[60] Rui W., Zhang D., Li S., Gao J., Han L., Qiu J. (2022). Simple Bioassay for PAMP-Triggered Immunity in Rice Seedlings Based on Lateral Root Growth Inhibition. Rice Sci..

[61] Meng F., Zhao Q., Zhao X., Yang C., Liu R., Pang J., Zhao W., Wang Q., Liu M., Zhang Z. (2022). A rice protein modulates endoplasmic reticulum homeostasis and coordinates with a transcription factor to initiate blast disease resistance. Cell Rep..

