# Supplementary material for: Ubiquitin Ligase Gene OsPUB57 Negatively Regulates Rice Blast Resistance
Source: Plants (Basel). 2025 Mar 1;14(5):758. doi: 10.3390/plants14050758 (PMC11901467; doi:10.3390/plants14050758)
Supplement: Supplementary file 1 [file plants-14-00758-s001.zip › plants-3397166-supplementary.pdf]

Table S1 Primer sequences used in this study

Primer name	Primer Sequence (5'-3')	Primer function
PUB57-qF	AAGCCACCGGACATACCAA	The amplification of *OsPUB57*
PUB57-qR	TGTTACGGCCTTTTCTTTCTGA	in qRT-PCR
BIERF3-F	CGGGCAACAAGCGGCCATAT	The amplification of *OsBIERF3*
BIERF3-R	AGACGACGACGAGGAGGAAGAGGA	in qRT-PCR
AOS2-F	TACCAGCCGTGCGCCACCAG	The amplification of *OsAOS2* in
AOS2-R	AGGACGGAGCTGGTTGAGTGG	qRT-PCR
PAL1-F	CCGACCACCTGACTCACAA	The amplification of *OsPAL1* in
PAL1-R	ATCTCACGCTCGATGGACTT	qRT-PCR
TSD2-F	TGGAAGTTGATCGCATCCTAC	The amplification of *OsTSD2* in
TSD2-R	CCACCTGAGCTGTGTTACAA	qRT-PCR
DREB1A-F	ATGTGCGGGATCAAGCAGGAGATG	The amplification *of OsDREB1A*
DREB1A-R	TGTCGAACGTGCCGAGCCAGAG	in qRT-PCR
Wrky45-F	GAATTCGGTGGTCGTCAAGA	The amplification of *OsWrky45*
Wrky45-R	GGAAGTAGGCCTTTGGGTG	in qRT-PCR
JAmyb-F	GAGGACCAGAGTGCAAAAGC	The amplification of *OsJAmyb* in
JAmyb-R	CATGGCATCCTTGAACCTCT	qRT-PCR
HLP1-F	GTATGGAGAAATTAAGCTAGC	The amplification of *OsHLP1* in
HLP1-R	AGTCCATGAAGAAAGCTGTC	qRT-PCR
Ub-qF	AACCAGCTGAGGCCCAAGA	The amplification of *Ubiquitin* in
Ub-qR	ACGATTGATTTAACCAGTCCATGA	qRT-PCR and biomass identification of *Magnaporthe oryzae*
MoPot2-F	ACGACCCGTCTTTACTTATTTGG	The amplification of *Pot2* in
MoPot2-R	AAGTAGCGTTGGTTTTTGTTGGAT	biomass identification of *Magnaporthe oryzae*
PUB57-ox-F	TATGCAAACCGGGTTCTCCAA	Overexpression vector
PUB57-ox-R	CTAGGTTTCTGACAACTGCT	construction
PUB57-TF1	GCCGCATTCGAGAATTGGAGAAC	The target adapter preparation for
PUB57-TR1	AAACGGTTCTCCAATTCTCGAATG	gene-editing vector construction
57-test-F1	ACCCCCTTGATGTCTCGTA	Target fragment amplification for
57-test-R1	TGGTATGTCCGGTGGCTTCAA	the genotype identification of gene-editing plants
U-F	CTCCGTTTTACCTGTGGAATCG	sgRNA expression cassette
gRNA-R	CGGAGGAAAATTCCATCCAC	amplification (first round) for gene-editing vector construction
Uctcg-B1	TTCAGAGGTCTCTCTCGCACTGGAATCGGCAGCAAAGG	sgRNA expression cassette amplification (second round) for gene-editing vector construction
gRcggt-BL	AGCGTGGGTCTCGACCGGGTCCATCCACTCCAAGCTC	

Table S2 The *cis*-acting elements on the promoter region of *OsPUB57*

Function	Name of element	Copy number	Position
Light responsive	G-box	1	891(-)
	GT1-motif	1	802(-)
	MNF1	1	1147(+)
	Sp1	2	68(-), 1174(+)
Fungal elicitor response	Box-W1	1	235(-)
Meristem expression	CAT-box	1	645(+)
JA signal transduction	CGTCA-motif	1	1301(+)
	TGACG-motif	1	1301(-)
GA signal transduction	GARE-motif	1	396(-)
Endosperm expression	GCN4-motif	1	830(-)
	Skn1-motif	2	105(+), 211(-)
Heat stress response	HSE	1	1031(+)
Low temperature	LTR	2	782(+), 1015(-)
Drought stress response	MES	1	272(-)
Defense and stress response	TC-rich repeats	2	493(-), 1102(-)

Table S3 The genotypes of target sites in three mutant lines of *OsPUB57*. The blue colored nucleotides indicate the target sequence of wild type. The red colored nucleotides indicate the protospacer adjacent motif (PAM). The dotted lines are the base deletions. A bold base represents the inserted base.

Mutant line	Genotype of target site	Mutant type
NPB (wild type)	CCGGGTTCTCCAATTCTCGAATG	
M-1	CCGGGTT**T**CTCCAATTCTCGAATG	+1bp
M-22	CCGGGT--TCCAATTCTCGAATG	-2bp
M-35	CCGGGT-CTCCAATTCTCGAATG	-1bp

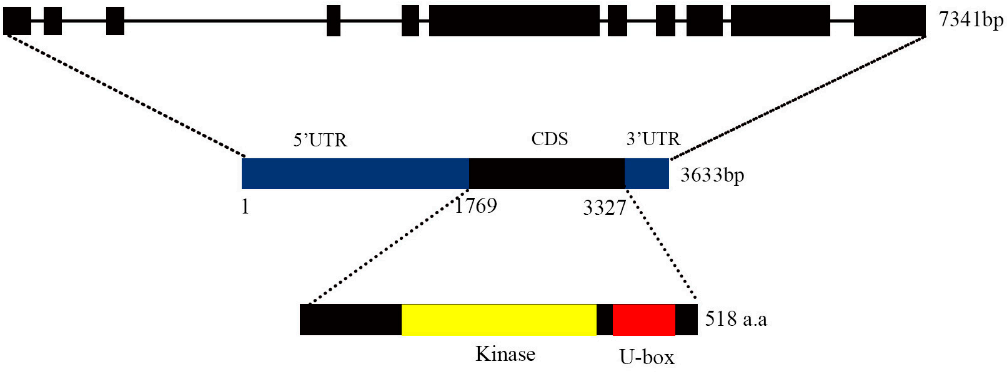

Figure S1 Schematic structure of *OsPUB57*

```
NPB      MQTGFSNSRMNSFISSVLVLHFNKQEIKFEPSVWCEAIDIHNTFSAGEIITGDIICFQKILKPPDIPKYPSVASFLQHVCDRKTYEEVRKVHILEEEIVT   100
M-1      MQTGFLQFSNEQFYLICTGFALQQAGNKI.......................................................................    29
M-22     MQTGFQFSNEQFYLICTGFALQQAGNKI........................................................................    28
M-35     MQTGSPILE...........................................................................................     9
Consensmqtg

NPB      LKHQADTYLVQKEKAVTAYDQLKHERDNAVQQVNELRDQSTHIILDFSRKDMEQATEHFKNAREVGDTEYGHTYKGMIHNMKVLIKLSSSQKLFQQEVSI   200
M-1      ....................................................................................................    29
M-22     ....................................................................................................    28
M-35     ....................................................................................................     9
Consensus

NPB      LRQWRHPNIITFIGVCSEVSALVYEWLPNGNLEDRIICTNNSAPLSWYNRTQIIGEICCALLFLHSNKSTALVHGDLRPCNILIDANYRSKICNFGMSNL   300
M-1      ....................................................................................................    29
M-22     ....................................................................................................    28
M-35     ....................................................................................................     9
Consensus

NPB      FLQLGTFPPNLTARLPYMDPEFNTTGELTTLSDVYSLGVIILRLLTGMPPLTLSEKVAEALGSDSLHLLIDKSAGDWPYIEAKQLALIGLSCTGMTRKKR   400
M-1      ....................................................................................................    29
M-22     ....................................................................................................    28
M-35     ....................................................................................................     9
Consensus

NPB      PDLLNEVWIVIEPLTRKPPAATWPYLQSASGDSSVPAAFICPISMEIMKDPQVASDGFTYEAEAIRCWFDRGISRSPMTNLALPNLNLVPNRVLRSFIHG   500
M-1      ....................................................................................................    29
M-22     ....................................................................................................    28
M-35     ....................................................................................................     9
Consensus

NPB      YLQQQQPNPAYQQQLSE                                                                                     517
M-1      .................                                                                                      29
M-22     .................                                                                                      28
M-35     .................                                                                                       9
Consensus
```

Figure S2 The amino acid sequence comparison of OsPUB57 in Nipponbare (NPB) and three mutants (M-1, M-22 and M-35).

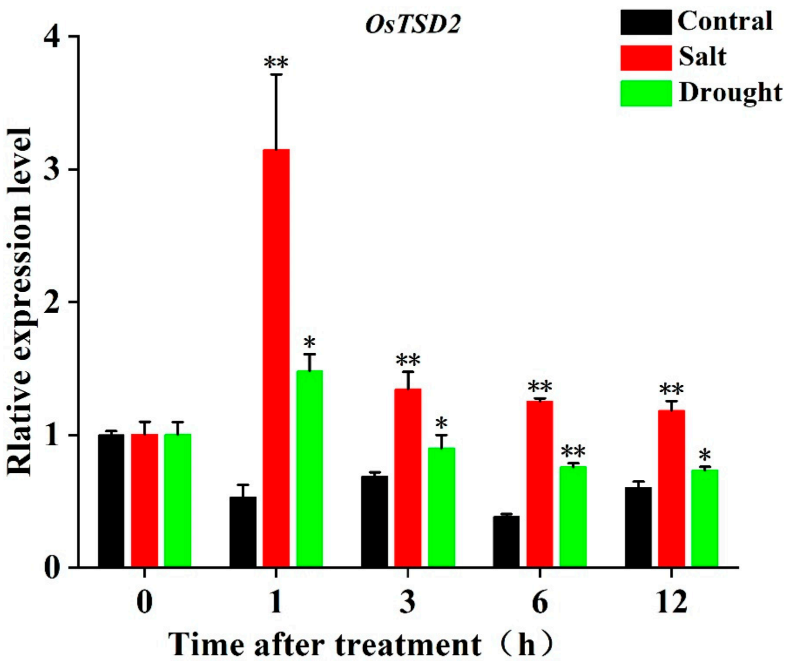

Figure S3 The transcriptional expression level of *OsTSD2* in rice under salt and drought treatments.

Note: Asterisks represent significant differences compared with the control. * represents p < 0.05, ** represents p < 0.01. The same below

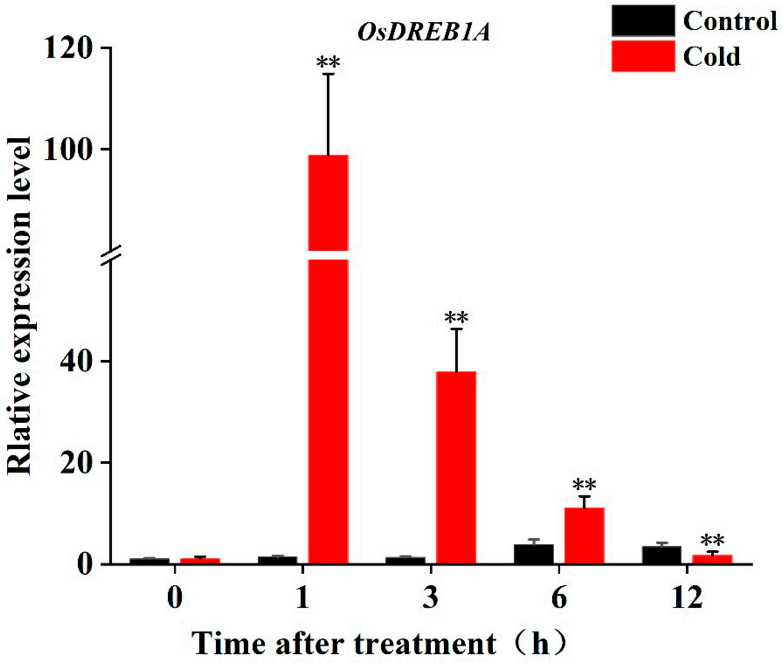

Figure S4 The transcriptional expression level of *OsDREB1A* in rice under cold treatment.

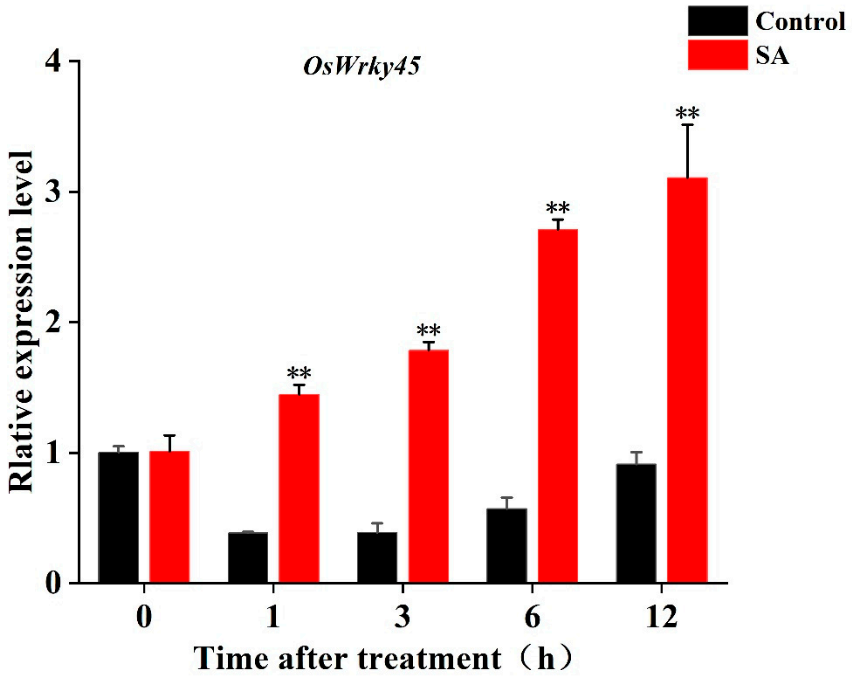

Figure S5 The transcriptional expression level of *OsWrky45* in rice under SA treatment.

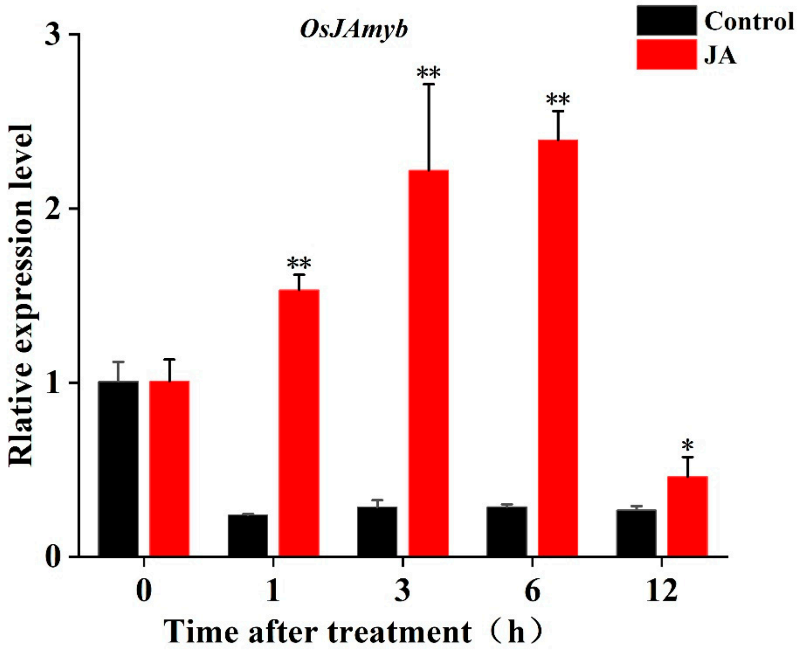

Figure S6 The transcriptional expression level of *OsJAmyb* in rice under JA treatment.

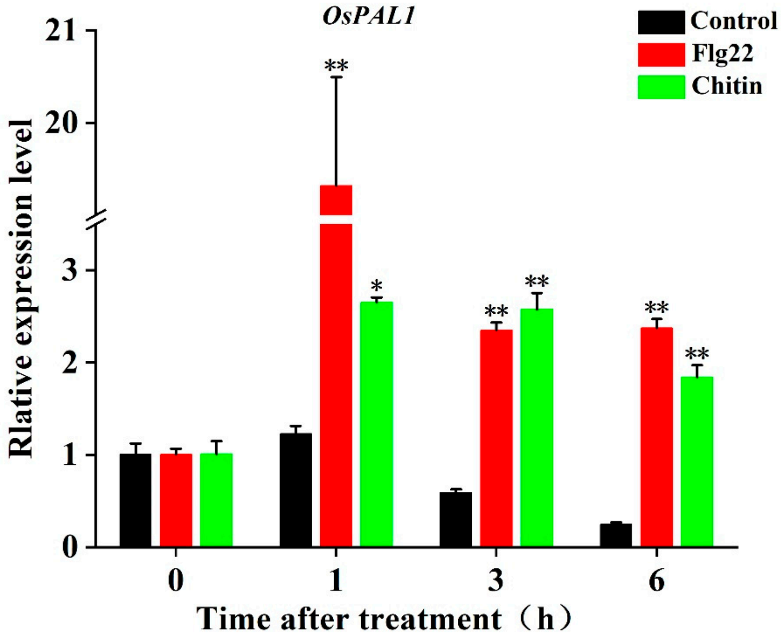

Figure S7 The transcriptional expression level of *OsPAL1* in rice under PAMP (flg22 or chitin) treatments.

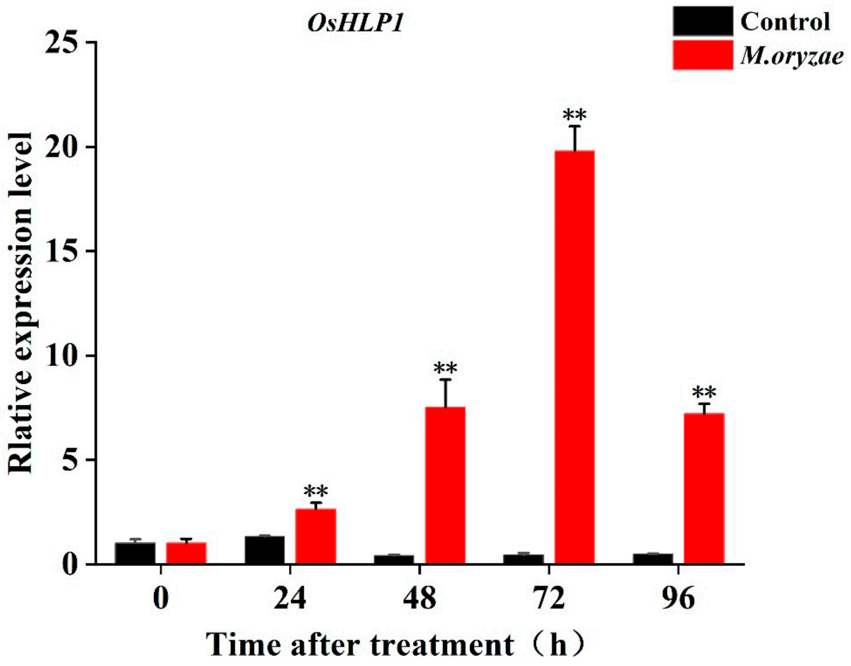

Figure S8 The transcriptional expression level of *OsHLP1* in rice after *M. oryzae* inoculation.
